# The relationship between the Ottawa Valve Collapse Scale (OVCS) and clinical outcomes in septoplasty patients

**DOI:** 10.1186/s40463-020-00407-8

**Published:** 2020-03-16

**Authors:** Phillip Staibano, James P. Bonaparte

**Affiliations:** 1 Faculty of Medicine, University of Ottawa, Ottawa, ON Canada; 2 Department of Otolaryngology—Head & Neck Surgery, University of Ottawa, 105A – 460 West Hunt Club Road, Ottawa, ON K2E 0B8 Canada

**Keywords:** Ottawa valve collapse scale (OVCS), Nasal obstruction, Nasal valve, Septoplasty, Patient outcomes

## Abstract

The Ottawa Valve Collapse scale (OVCS) was developed to classify the severity of nasal valve collapse (NVC) in patients with nasal obstruction. The goal of this study was to determine, in patients who have nasal obstruction due to a septal deviation, whether those with a higher OVCS grade will have a reduced improvement in patient-centered clinical outcomes at one-year following septoplasty with inferior turbinate diathermy compared to those with a normal or lower OVCS grade. This study was a prospective study of 78 patients who completed an assessment using the NOSE questionnaire before and at one-year following the surgical intervention. A repeated-measures ANOVA was used to asses for differences in scores between OVCS groups. There was a significant improvement in NOSE scores one year post-septoplasty (*p* < 0.01). There was no difference in NOSE score improvement when comparing the grades of the OVCS at one-year (F = 0.09, *p =* 0.968). Though the OVCS was designed to categorize the severity of NVC preoperatively, there is no evidence that it is helpful in predicting which patients will demonstrate poor results following septoplasty. Future studies are required to further evaluate the OVCS and whether complimentary assessments will improve its clinical utility.

## Introduction

Nasal airway obstruction is a common patient complaint in otolaryngology practice [1, 2]. Septal deviation leading to chronic nasal airway obstruction can reduce patient wellbeing [3]. Surgical repair of septal deviation is associated with a reversal of obstructive symptoms and improvements in quality of life [4, 5]. However, dissatisfaction rates following septoplasty remain as high as 51%, which suggests that in some patients the underlying cause of nasal obstruction may be improperly diagnosed prior to surgery [6].

Nasal valve collapse (NVC), which occurs in the presence of decreased intraluminal pressure within the nasal cavity, can exacerbate airway obstruction [7]. In addition to septal deviation, NVC can develop in the context of other anatomical abnormalities, including lateral wall insufficiency, wide columella, and hypertrophied inferior turbinate [8]. Currently, however, no single diagnostic investigation can reliably distinguish between the underlying causes of NVC: an obstacle that may impede proper preoperative assessment of patients with airway obstruction.

The Cottle manoeuver remains a common physical examination technique for assessing NVC, whereby the patient is asked to inspire while the physician applies tension to the internal nasal valve [9]. The Cottle maneuver and modified Cottle maneuver are limited in their ability to evaluate NVC severity [10]. Currently, there is lack of agreement on a gold standard diagnostic test in evaluation of NVC and perhaps more importantly, predicting when NVC is clinically significant and requiring repair [7]. In response to these limitations, our team developed the Ottawa Valve Collapse Scale (OVCS), which uses an ordinal measurement scale to grade the severity of external nasal valve collapse without altering nasal anatomy [11]. The OVCS has demonstrated good test-retest reliability and construct validity, but requires further investigation in determining whether it can predict patients who will benefit most from septoplasty and which patients may have a higher risk of surgical failure. In addition to the OVCS, the Nasal Obstruction Symptom Evaluation (NOSE) scale evaluates the impact of airway obstruction on quality of life [12]. The objective of this study was to determine, in patients with a septal deviation, whether a high preoperative OVCS grade is associated with a worsened NOSE outcomes one year following septoplasty with inferior turbinate diathermy.

## Materials and methods

### Study design

We performed a prospective study of patients who attended the community practice of primary investigator/physician (JB). JB is a member of the Department of Otolaryngology–Head and Neck Surgery at The Ottawa Hospital. This study was approved by the Ottawa Health Science Network Research Ethics Board.

### Patient population

All patients included in this study were at least 18 years old and presented to the private otolaryngology clinic of the author (JB) with subjective nasal obstruction. All included patients had been diagnosed with clinically symptomatic septal deviation with or without NVC and underwent septoplasty with inferior turbinate diathermy. All patients underwent a cosmetic septoplasty rather than a functional procedure due to a lack of severe nasal obstruction symptomatology. Patients included in this study completed the NOSE questionnaire and had their nasal valve graded using the OVCS prior to and following septoplasty. Patients were excluded if they had any prior nasal surgery, a known traumatic cause of their septal deviation, a history of allergic rhinitis, chronic rhinosinusitis, neoplastic or autoimmune processes causing nasal obstruction, a septal perforation, and/or a fixed nasal valve resulting in narrowing not related to a septal deviation (i.e. prior cleft lip repair).

### Ottawa Valve Collapse Scale (OVCS)

Our team has developed a four-grade scoring tool, known as the OVCS, to grade nasal valve collapse [11]. According to the OVCS grading scale, a zero is defined as no external valve collapse. A grade of one is defined as unilateral partial valve collapse [i.e., an active narrowing of the external nasal valve occurring during deep inspiration without complete airflow blockage (≤99%)]. A grade of two is defined either bilateral partial collapse (≤99%) or unilateral complete collapse [i.e., complete airflow blockage and nasal side alar or lower lateral side mucosa contacting the septum medially (100%)]. A grade of three is defined as complete bilateral nasal valve collapse (100%).

### Nasal obstruction symptom evaluation (NOSE) scale

The NOSE scale is a validated, patient-completed 5-item subjective instrument that evaluates the impact of nasal obstruction on quality of life [12]. These 5 items include nasal congestion or stuffiness, nasal blockage or obstruction, trouble breathing through nose, trouble breathing, and trouble getting enough air through nose during exercise or exertion. Each item is scored on a 5-point Likert scale to make a total score between 0 and 100, with higher scores indicating worsened nasal obstruction symptoms.

### Study protocol and study outcomes

All patients in this study had their nasal valve collapse graded by the OVCS prior to septoplasty. For each patient, three trials of deep inspiration were elicited, with the highest score being chosen as the OVCS grade for that individual patient. OVCS grading was performed by Dr. J. Bonaparte. We also recorded the result of the Cottle maneuver prior to septoplasty. In addition, all patients completed the NOSE scale at two time points, before surgery and at 12 months following surgery. The total NOSE score was used as the outcome measure at both time points. For each patient, we also recorded demographic variables, including age and gender, and the time lapse since septoplasty.

### Statistical analyses

All patients were stratified into four groups based on OVCS grade (i.e. 0–3) prior to septoplasty. A Kruskal-Wallis Test was employed to compare baseline NOSE scores and preoperative OVCS grades due to the lack of distribution normality. We performed a repeated-measures ANOVA to compare postoperative total NOSE scores across preoperative OVCS stratification groups. Standard deviation (SD) was employed as measure of error. Statistical significance was defined as a *p* < 0.05.

## Results

In total, 78 patients fulfilled all eligibility criteria and completed the study. Mean age of all patients was 40.8 (SD: 13.3). The majority of patients included had a preoperative OVCS grade of 0 or 1 (Table 1). Mean baseline NOSE score for all patients was 65.9 (SD: 3.81). Mean baseline NOSE score stratified by preoperative OVCS grade is shown in Table 2. A Kruskal-Wallis test of ranks demonstrated that pre-operative NOSE scores at baseline did not differ significantly when patients were stratified based on preoperative OVCS grade (H = 3.23, *p* = 0.357).

**Table 1:** Baseline demographic characteristics (*N* = 78)

OVCS grade	No. of patients (%)	Mean age (SD)	No. female (%)
0	32 (41)	42.3 (12.6)	12 (37.5)
1	25 (32.1)	37.2 (13)	11 (44)
2	15 (19.2)	39.5 (12.8)	8 (53.3)
3	6 (7.69)	50.9 (16.4)	2 (33.3)

**Table 2:** Mean NOSE score at one-year post-septoplasty stratified by preoperative Ottawa Valve Collapse Scale (OVCS) grade

OVCS grade	Baseline NOSE score (SD)	One-year NOSE score (SD)
0	70 (3.71)	23.2 (5.17)
1	61 (3.75)	22.2 (4.10)
2	62.5 (4.12)	23.7 (5.34)
3	70 (3.46)	20 (2.19)

*p* > 0.9

Mean NOSE scores at one-year post-septoplasty as stratified by preoperative OVCS are presented in Table 2. For all 78 patients, we found that mean NOSE score at one-year post-septoplasty improved significantly when compared to mean preoperative NOSE score (22.7 vs. 65.9, respectively, *p < 0.01).* A repeat-measures ANOVA demonstrated that the one-year post-septoplasty NOSE score did not differ significantly across OVCS grades (F = 0.09, *p =* 0.968).

## Discussion

Misclassification of the etiology of nasal obstruction prior to surgery is a factor in patient dissatisfaction following septoplasty [6]. The OVCS was developed to evaluate the degree of NVC severity without altering nasal anatomy. The OVCS has been shown to have good test re-test reliability and construct validity [11], but until this study, was not evaluated for clinical utility.

Here, we demonstrate that the severity of NVC, as denoted by higher preoperative OVCS grades, was not associated with worsened quality of life one year following septoplasty with inferior turbinate diathermy nor was the OVCS associated with more severe preoperative symptomatology. This suggests that this type of stratification is not effective at predicting who may fail a septoplasty due to NVC. A recent study of the effectiveness of the Cottle maneuver in predicting outcomes one-year following septoplasty demonstrated that a positive preoperative Cottle maneuver was not associated with worsened postoperative NOSE outcomes [13]. Other tools are also currently being studied to grade the severity of NVC: the NAISON assessment, which combines history, physical examination, allergy testing, and maximum-effort flow-volume loops to characterize NVC severity and has been shown to improve septoplasty outcomes [14], and a standardized nasal anatomic worksheet that has been shown to correlate with symptoms of nasal obstruction [15]. Moreover, a recent study has shown that peak nasal inspiratory flow is associated with nasal obstruction and NOSE scores, and may therefore be a useful factor in the comprehensive appraisal of nasal obstruction [16]. Therefore, although this study suggests that the OVCS in its current form is not useful in predicting post-septoplasty functional outcomes, the predictive utility of OVCS might be improved by the addition of complementary assessments, including peak nasal inspiratory flow, to characterize NVC severity.

Additional tools, such as SNOT-22 [17], also demonstrate utility in evaluating the patient-centred outcomes of nasal obstruction and septoplasty [18]. Other subjective tools, including the Glasgow Benefit Index have demonstrated improvement following septoplasty [19], and objective measures, including acoustic rhinometry are effective in evaluating septoplasty outcomes [20]. Findings have also suggested that psychological factors and personality characteristics contribute to differences in the perception of septoplasty outcomes [21]. Studies are now demonstrating that evaluating a compliment of functional, aesthetic, and subjective outcomes may help to better capture the full impact of NVC severity and septoplasty on patient-centred outcomes.

We also demonstrate a significant improvement in NOSE scores among all patients one year following septoplasty in patients with and without evidence of NVC. These findings are consistent with other studies demonstrating that the NOSE tool is able to identify clinical improvements following septoplasty and functional rhinoplasty [22, 23]. Baseline NOSE scores did not differ significantly when stratified by preoperative OVCS grade. This is in contrast with recent findings demonstrating that increased OVCS severity was associated with worsened nasal obstruction symptomatology [11]. Limitations of the current study include the small cohort size and the limited number of patients who scored a preoperative OVCS grade of ≥2. A lack of severe OVCS grading may have contributed to the similar NOSE scores within this patient cohort and, therefore, an inability to apply these conclusions to patients with severe NVC. Another limitation is that post-septoplasty outcomes in this study were only evaluated using the NOSE tool, but NOSE scores can be influenced by other patient-based factors [24, 25].

In summary, these findings suggest that preoperative OVCS grade is not predictive of patient-centred outcomes one year following septoplasty. Future studies are required to determine the utility of the OVCS in patients with severe NVC and whether preoperative OVCS grading requires the addition of other evaluations to optimize clinical utility.

## Data Availability

All material is available for review.

## Abbreviations

OVCS: Ottawa Valve Collapse Scale
NVC: Nasal Valve Collapse

## References

[1] Reitzen SD, Chung W, Shah AR (2011). Nasal septal deviation in the pediatric and adult populations. Ear Nose Throat J.

[2] Lipan MJ, Most SP (2013). Development of a severity classification system for subjective nasal obstruction. JAMA Facial Plast Surg.

[3] Udaka T, Suzuki H, Kitamura T (2006). Relationships among nasal obstruction, daytime sleepiness, and quality of life. Laryngoscope.

[4] Manoukian PD, Wyatt JR, Leopold DA (1997). Recent trends in utilization of procedures in otolaryngology-head and neck surgery. Laryngoscope.

[5] Bugten V, Nilsen AH, Thorstensen WM (2016). Quality of life and symptoms before and after nasal septoplasty compared with healthy individuals. BMC Ear Nose Throat Disord.

[6] Konstantinidis I, Triaridis S, Triaridis A (2005). Long term results following nasal septal surgery. Focus on patients’ satisfaction. Auris Nasus Larynx.

[7] Rhee JS, Weaver EM, Park SS (2010). Clinical consensus statement: diagnosis and management of nasal valve compromise. Otolaryngol Head Neck Surg.

[8] Simon P, Sidle D (2012). Augmenting the nasal airway: beyond septoplasty. Am J Rhinol Allergy.

[9] Fung E, Hong P, Moore C (2014). The effectiveness of modified cottle maneuver in predicting outcomes in functional rhinoplasty. Plast Surg Int.

[10] Gruber RP, Lin AY, Richards T (2011). Nasal strips for evaluating and classifying valvular nasal obstruction. Aesthet Plast Surg.

[11] Ziai H, Bonaparte JP (2018). Reliability and construct validity of the Ottawa valve collapse scale when assessing external nasal valve collapse. J Otolaryngol Head Neck Surg.

[12] Stewart MG, Witsell DL, Smith TL (2004). Development and validation of the nasal obstruction symptom evaluation (NOSE) scale. Otolaryngol Head Neck Surg.

[13] Bonaparte JP, Campbell R (2018). A prospective cohort study assessing the clinical utility of the Cottle maneuver in nasal septal surgery. J Otolaryngol Head Neck Surg.

[14] Nouraei SA, Virk JS, Kanona H (2016). Non-invasive assessment and symptomatic improvement of the obstructed nose (NASION): a physiology-based patient-centred approach to treatment selection and outcomes assessment in nasal obstruction. Clin Otolaryngol.

[15] Lindsay RW, George R, Herberg ME (2016). Reliability of a standardized nasal anatomic worksheet and correlation with subjective nasal airway obstruction. JAMA Facial Plast Surg.

[16] Fuller JC, Gadkaree SK, Levesque PA (2019). Peak nasal inspiratory flow is a useful measure of nasal airflow in functional septorhinoplasty. Laryngoscope.

[17] Laababsi R, Abdulhakeem B, Elkrimi Z (2019). Quality of life outcomes of patients with chronic rhinosinusitis after functional endoscopic sinus surgery, prospective cohort study. Ann Med Surg.

[18] Kordjian HH, Schousboe LP (2017). Sixty-three patient-based survey-can Sino-nasal outcome Test-22 be a suitable evaluation method for septoplasty and turbinectomy?. Clin Otolaryngol.

[19] Valsamidis K, Titelis K, Rachovitsas D (2018). Long-term evaluation of nasal Septoplasty followed by inferior turbinate cauterization for the treatment of nasal obstruction using objective and subjective methods. Int Arch Otorhinolaryngol.

[20] Tiftikcioglu YO, Gur E, Songur E (2017). Nasal functional evaluation using nasal endoscopy, acoustic Rhinometry, and Rhinomanometry on nasal airway-obstructed patients after endoscopic Septoplasty, corrective Rhinoplasty, and internal nasal valve surgery. Turkish J Plast Surg / Türk Plastik Rekonstrüktif ve Estetik Cerrahi Dergisi.

[21] Saragusty C, Berant E, Yaniv E (2011). Association of attachment anxiety and satisfaction with nasal surgery. Rhinology.

[22] Kahveci OK, Miman MC, Yucel A (2012). The efficiency of NOSE obstruction symptom evaluation (NOSE) scale on patients with nasal septal deviation. Auris Nasus Larynx.

[23] Most SP (2006). Analysis of outcomes after functional rhinoplasty using a disease-specific quality-of-life instrument. Arch Facial Plast Surg.

[24] Rozanski C, Gray M, Rousso JJ (2019). Disparities in NOSE scores and surgical approaches among patients undergoing functional Rhinoplasty. Facial Plast Surg.

[25] Valsamidis K, Titelis K, Karkos P (2019). Predictive factors of patients’ general quality of life after nasal septoplasty. Eur Arch Otorhinolaryngol.

